# Brusatol suppresses meningioma progression via targeting HMGCR to restrict the cholesterol biosynthesis and inhibit PI3K/AKT signaling pathway

**DOI:** 10.3389/fphar.2026.1864031

**Published:** 2026-06-30

**Authors:** Huaning Li, Wei Xie, Xing Cheng, Wen Shen, Run Shi, Zhumei Shi, Zhichao Wang, Yu Gao, Xiefeng Wang, Mengjie Guo, Yongping You

**Affiliations:** 1 Department of Neurosurgery, The First Affiliated Hospital of Nanjing Medical University, Nanjing, China; 2 Institute for Brain Tumors, Jiangsu Key Lab of Cancer Biomarkers, Prevention and Treatment, Jiangsu Collaborative Innovation Center for Cancer Personalized Medicine, Nanjing Medical University, Nanjing, China; 3 Department of Neurosurgery, Jiangsu Province (Suqian) Hospital, Suqian, China; 4 School of Pharmacy, Nanjing University of Chinese Medicine, Nanjing, China; 5 School of Medicine & Holistic Integrative Medicine, Nanjing University of Chinese Medicine, Nanjing, China

**Keywords:** brusatol, cholesterol biosynthesis, HMGCR, meningioma, PI3K/akt signaling pathway

## Abstract

**Background:**

3-hydroxy-3-methylglutaryl-CoA reductase (HMGCR), a key enzyme in cholesterol metabolism, remains underexplored in meningioma. Additionally, the therapeutic potential of Brusatol (Bru), a triterpene lactone compound with anticancer properties, has yet to be systematically evaluated. This research investigates Bru’s efficacy in meningioma treatment and HMGCR-related mechanisms.

**Methods:**

IC_50_ was determined using CCK-8 assay. Proliferation, apoptosis, migration, and invasion were assessed through colony formation, EdU, Annexin V/PI staining, Scratch, and Transwell assays, respectively. Cholesterol levels were measured with an assay kit and Filipin III probes. Key pathways and potential meningioma-specific marker were identified via multi-omics with GO/KEGG analyses and bioinformatic analyses. Protein expression was detected via Western blot analysis, and a mouse model of meningioma was employed to evaluate the efficacy of Bru.

**Results:**

Bru inhibited the proliferation, migration, and invasion of meningioma cells, induced apoptosis, and reduced cholesterol accumulation. Proteomic, transcriptomic, and bioinformatic analyses, along with molecular docking, revealed that Bru targeted HMGCR to restrict cholesterol biosynthesis and inhibit the PI3K/AKT signalling pathway. These findings were validated through a series of experiments, including surface plasmon resonance assay, HMGCR knockdown and overexpression, and rescue experiments where HMGCR knockdown or Bru treatment was reversed with an Akt activator SC79. *In vivo* experiments indicated that Bru effectively suppresses tumour progression, with no significant toxicity observed under the current experimental conditions.

**Conclusion:**

These findings demonstrate that Brusatol suppresses meningioma progression by inhibiting of HMGCR and the PI3K/AKT signaling pathway.

## Graphical Abstract

**Figure:**
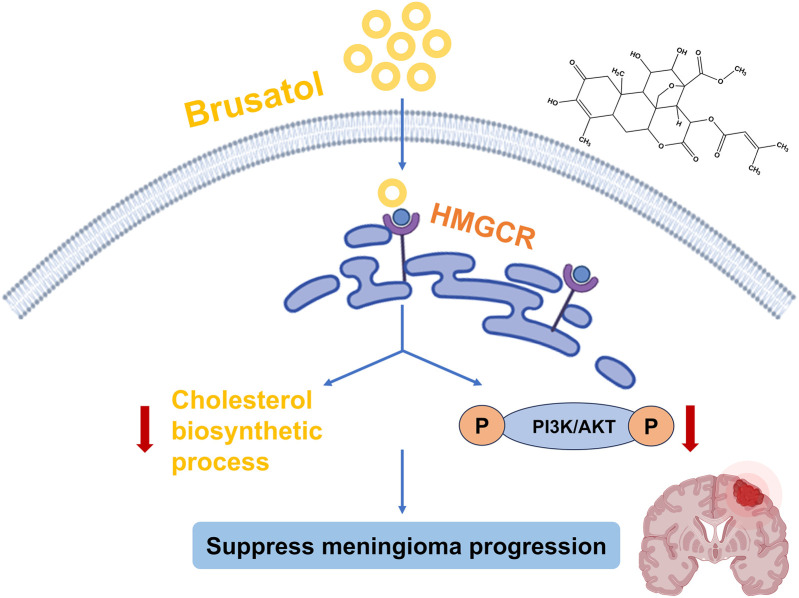

## 1 Introduction

Meningioma is the most frequently diagnosed primary tumour of the central nervous system, with a high incidence rate of approximately 7.61 cases per 100,000 individuals (Ostrom et al., 2015). Clinicians preferentially perform maximal safe resection of this brain tumour, followed by adjuvant radiotherapy, especially for high-grade meningioma (Holleczek et al., 2019). However, the high recurrence rate of high-grade meningioma is associated with a median overall survival of merely 4 years (Goldbrunner et al., 2016). Expansion of therapeutic options via systemic therapies is therefore urgently required for these patients. To date, over 20 distinct agents, including hydroxyurea, temozolomide, tamoxifen, trabectedin, and various tyrosine kinase inhibitors, have been clinically evaluated. In the majority of these studies, which were small-scale and non-randomized, these agents did not exhibit significant clinical benefits in prolonging survival, reducing tumour recurrence, or improving the quality of life in patients with meningioma (Jungwirth et al., 2023; Goldbrunner et al., 2021). An effective and efficient systemic therapeutic strategy targeting novel pathogenic mechanisms has not yet been well established for this patient population.

Metabolic reprogramming, particularly of cholesterol metabolism, which plays a vital role in tumour progression, has recently garnered extensive attention in the realm of cancer therapy (Xiao et al., 2023). Cholesterol is essential not only for preserving membrane fluidity and electrical properties but also facilitates the proliferation of tumour cells by modulating multiple signalling pathways and affecting membrane synthesis (Patel and Kashfi, 2022). Abnormal accumulation of cholesterol correlates with an array of oncogenic processes, such as tumour cell growth, metastasis, cancer stem cell development, angiogenesis, and resistance to treatment in multiple cancer types (Kuzu et al., 2016; Xue et al., 2020). Consequently, the dysregulation of cholesterol metabolism is essential for driving cancer progression and represents a potential therapeutic target. However, despite the fact that these findings have been validated in various types of cancers, the specific alterations in cholesterol metabolism associated with meningioma progression remain to be clarified.

*De novo* cholesterol biosynthesis occurs predominantly within the endoplasmic reticulum via the mevalonate pathway (Xu et al., 2020). Among the enzymes catalysing the approximately 30 enzymatic reactions involved in the biosynthesis, 3-hydroxy-3-methylglutaryl-CoA reductase (HMGCR) is as a crucial enzyme that regulates the intracellular cholesterol synthesis rate (Xue et al., 2020). The elevated expression of HMGCR in a range of malignancies, such as stomach, bladder, liver, and prostate cancers, enhances the aggressive phenotypes of cancer cells (Jiang et al., 2024). Despite their promise, current therapeutic strategies targeting cholesterol metabolism, such as those using enzyme inhibitors and modulators of cholesterol transport proteins, face significant challenges including adverse side effects, development of drug resistance, and limited sustained efficacy in a large population of patients (Bell et al., 2024). This underscores the urgent need for devising novel therapeutic approaches to address these challenges.

**FIGURE 1:**
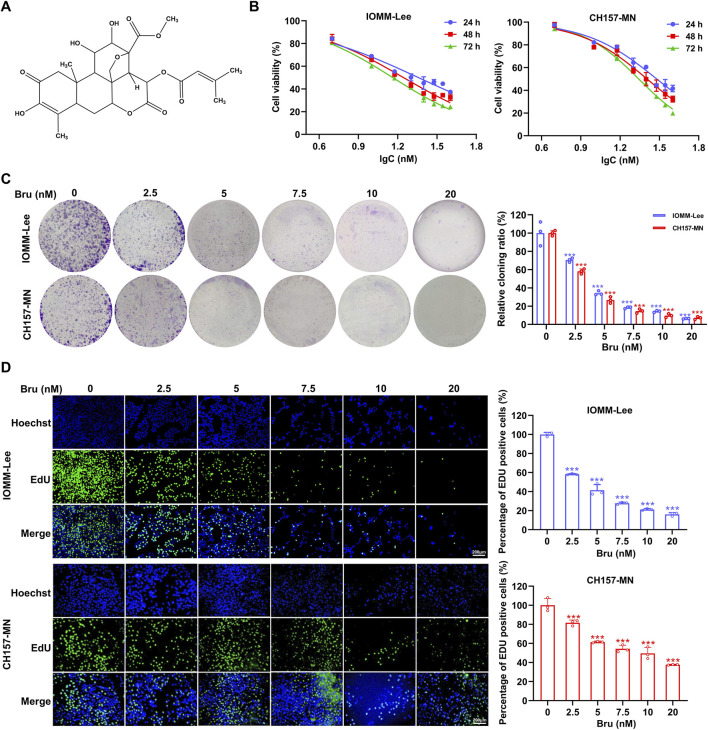
Brusatol (Bru) inhibited the proliferation of meningioma cell lines **(A)** Chemical structure of Bru **(B)** Dose-response curves of Bru in IOMM-Lee and CH157-MN cells generated using the cell counting kit-8 assay **(C)** Effect of Bru on the colony-forming ability of meningioma cells, assessed using the colony formation assay **(D)** Inhibition of the proliferation of IOMM-Lee and CH157-MN cells by Bru, as evaluated using the five-ethynyl-2′-deoxyuridine (EdU) assay. Data are presented as mean ± SD (*n* = 3). ^*^ *p* < 0.05; ^**^ *p* < 0.01; ^***^ *p* < 0.001 vs. the control group.

Traditional Chinese medicine (TCM) has gained recognition as a promising treatment option for retarding tumour development, mainly owing to its multi-target regulatory mechanisms (Lin et al., 2017). Furthermore, numerous compounds derived from TCM have been noted for their low toxicity and natural origins, providing a robust scientific rationale for their use in cancer therapies (Ma et al., 2023). Brusatol (Bru, CAS: 14,907–98–3), the chemical structure is shown in Figure 1A — isolated from the seeds of *Brucea javanica* (L.) Merr, a Chinese medicinal herb traditionally used for treating inflammation, dysentery, malaria, and cancer—has a notable concentration of 0.3% (Zhou et al., 2011). An increasing number of studies have uncovered that Bru exhibits prominent anti-tumor effects across various types of tumors, such as pancreatic cancer, hepatocellular carcinoma, colorectal cancer, and lung cancer, with IC_50_ values preferentially below 100 nM in most tumor cell lines. Compared to other potential anti-tumor agents, Bru harbors unique advantages. It exhibits a wide range of anti-tumor activities and robust chemosensitizing functions across different types of cancers through regulating several signaling pathways, such as Nrf2 and PI3K/Akt/mTOR signaling cascade, as well as critical biological processes including epithelial-mesenchymal transition (EMT). Additionally, Bru has been demonstrated to enhance radiosensitivity and reverse chemoresistance (He et al., 2023). When employed in conjunction with chemotherapeutic agents, Bru not only boosts their anti-tumor effectiveness but also alleviates chemotherapy resistance via promoting ferroptosis and preventing the occurrence of EMT (Xi et al., 2024). Recent findings derived from the cellular and animal studies indicate that Bru may exert its anti-tumor properties through regulating the lipid metabolic pathways, with especially notable results in the models of hepatocellular carcinoma (Wan et al., 2024). In clinical settings, an emulsion preparation of B. javanica oil, with Bru as the major bioactive component, has been utilized alongside irinotecan for cancer treatment (Song et al., 2024). Furthermore, Bru in combination with other chemotherapeutic drugs has been documented to enhance chemotherapy safety, improve therapeutic outcomes, and foster better patient adherence (Wang et al., 2021). Nonetheless, to date, there has been no comprehensive investigation into Bru’s anti-tumor mechanisms with regard to targeting HMGCR and cholesterol biosynthesis pathways, nor have there been detailed clinical and preclinical studies evaluating its therapeutic effects against meningioma. Consequently, further studies into the effects of Bru on meningioma is of paramount importance.

The present study was aimed at investigating the therapeutic effectiveness of Bru on the development of meningioma. The research focused on clarifying the mechanisms of action of Bru, with a particular emphasis on inhibiting HMGCR to modulate the cholesterol biosynthesis and PI3K/AKT signalling pathway. This study, for the first time, explored the therapeutic potential of Bru in meningioma, and unravelled the underlying molecular mechanisms involved.

## 2 Materials and methods

### 2.1 Reagents

Bru was procured from Xinyang Zhongjian Measurement Biological Technology Co., Ltd (Xinyang, China). Fetal bovine serum (FBS) was obtained from VivaCell BIOSCIENCES (Shanghai, China). Dimethyl sulfoxide (DMSO) was sourced from MACKLIN (Shanghai, China). Dulbecco’s modified Eagle medium (DMEM) was purchased from Gibco (Suzhou, China). The Cell Counting Kit-8 (CCK-8), Amplex Red Cholesterol and Cholesterol Esters Test Kit, and fluorescein isothiocyanate (FITC) Annexin V Apoptosis Detection Kit were obtained from Beyotime (Shanghai, China). Antibodies against GAPDH, AKT, and PI3K were acquired from Proteintech (Wuhan, China), and those specific to phosphorylated AKT (p-AKT) and PI3K (p-PI3K) were sourced from Abcam (Shanghai, China). The HMGCR antibody was obtained from Abmart (Shanghai, China).

### 2.2 Cell culture

The IOMM-Lee and CH157-MN cell lines were provided by Professor Ji Jing from Nanjing Medical University and have been maintained in our laboratory. The IOMM-Lee cell line authenticated by short tandem repeat (STR) profiling (Shanghai Yihe Applied Biotechnology Co., Ltd., 2023) and matched the reference profile in the EXPASY database (CVCL_5779). CH157-MN was authenticated by short tandem repeat (STR) profiling (Suzhou Haixing Biotechnology Co., Ltd., 2024) All cell lines were tested negative for *mycoplasma* contamination by PCR assays before use. These cell lines were maintained in DMEM supplemented with 10% FBS and 1% penicillin-streptomycin (Solarbio, Beijing, China), incubated at 37 °C within a humidified environment containing 5% CO_2_. Before treatment, the cells reached approximately 80% confluence.

### 2.3 Assessment of cell viability

A stock solution of Bru (20 mM) was prepared in DMSO and subsequently diluted using sterile phosphate-buffered saline (PBS) or culture medium prior to treatment to obtain the required concentrations. The final concentration of DMSO was kept equal to or less than 0.1%, to match the concentration in the vehicle control group. IOMM-Lee and CH157-MN cells were plated in 96-well plates at a density of 5 × 10^3^ cells per well and treated with 0, 2.5, 5, 7.5, 10, and 20 nM different concentrations of Bru for 24, 48, and 72 h, respectively. The concentration range of Bru was determined based on preliminary cytotoxicity experiments in the two meningioma cell lines. Following the treatment, cell viability was evaluated using the CCK-8 assay. CCK-8 reagent was added at 10 μL per well, incubated for 3 h, and absorbance was measured at 450 nm. A concentration–response curve was subsequently generated on the basis of the obtained results.

### 2.4 Colony formation and five-ethynyl-2′-deoxyuridine (EdU) assays

IOMM-Lee and CH157-MN cells were seeded in six-well plates at a density of 1,500–2000 cells per well and cultured in complete medium supplemented with either 0.1% DMSO (vehicle control) or Bru. The medium containing fresh 0.1% DMSO or Bru was replaced every 2 days throughout the incubation period, after which the cells were continuously cultured for a total of 14 days. The resultant colonies were fixed with 4% paraformaldehyde and stained with 0.1% crystal violet. Images of the colonies were captured to assess the cell survival.

For the EdU assay, IOMM-Lee and CH157-MN cells were seeded in 24-well plates and incubated with different concentrations of Bru for 48 h. Thereafter, 20 μM EdU reagent was added according to the manufacture’s protocol. Subsequently, the cells were fixed, permeabilised, and subjected to the Click reaction. Fluorescence signals corresponding to EdU (green) and Hoechst (blue) were recorded using a fluorescence microscope.

### 2.5 Cell apoptosis analysis

IOMM-Lee and CH157-MN cells were seeded in six-well plates and incubated with different concentrations of Bru or DMSO. After 48-h of incubation, the cells were rinsed with PBS to eliminate any residual medium and cellular debris, trypsinised, collected via centrifugation, and stained using Annexin V-FITC and propidium iodide (PI). The stained cells were subsequently analysed on a flow cytometer.

### 2.6 Scratch and transwell invasion assays

IOMM-Lee and CH157-MN cells, maintained under appropriate growth conditions, were evenly seeded into six-well plates. Once the cells reached 100% confluence, a vertical scratch was made in the centre of each well using a 10-μL pipette tip. Subsequently, the medium was replaced with serum-free DMEM containing either DMSO or different concentrations of Bru. The scratched regions were examined and photographed under a microscope at the initial time point (0 h) and after 48 h of treatment. The dimensions of the scratches were measured using the ImageJ software.

For the Transwell invasion assay, the inserts were coated with Matrigel and then incubated at 37 °C for 3 h. Cell suspensions were prepared at a density of 2 × 10^5^ cells/mL for various treatment groups with 0, 2.5, 5, 7.5, 10, or 20 nM Bru. Subsequently, the cells were transferred into the upper Transwell chambers. The lower chambers were saturated with a culture medium enriched with 10% FBS, which acted as a chemoattractant. Following a 48-h incubation period, the migration of cells to the surface of the lower membrane was quantified.

### 2.7 Proteomic and transcriptomic analyses

Three parallel groups of IOMM-Lee cells were used for total protein extraction, quality assessment, and identification: control and Bru-treated groups (treated with 10 nM Bru for 48 h). Proteomic analysis was performed to identify differentially expressed proteins (DEPs), adhering to the criteria of |log2FC| ≥0.585 and a false discovery rate (FDR) ≤0.05. Data interpretation was conducted with the Kyoto encyclopedia of genes and genomes (KEGG) and gene ontology (GO) databases.

Following the standard protocol provided by the manufacturer, IOMM-Lee cells from both the experimental groups (control and Bru-treated) were used for transcriptomic analysis (*n* = 3). High-throughput RNA sequencing was carried out using the NovaSeq6000 platform. Data analysis was conducted utilizing DEGseq, whereby differentially expressed sequences (DESs) were discerned according to the parameters of |log2FC| ≥0.585 and FDR ≤0.05 (Li et al., 2025; Hu et al., 2025).

### 2.8 Gene correlation and protein interaction analysis

A correlation analysis of pivotal genes was performed using the SRplot platform (https://www.bioinformatics.com.cn/). The Pearson correlation coefficients for the identified significant genes were computed, and the corresponding correlation visualisations were generated via the SRplot platform.

Protein–protein interactions were assessed using the STRING database (https://string-db.org/). A list of significant genes was input into STRING, where only interactions with a combined score ≥0.9 were retained, whereas unconnected proteins were excluded from the visual representation. The resultant interaction network was then imported into Cytoscape (v3.9.1) for enhanced visualisation and subsequent analysis.

### 2.9 Molecular docking

The 3D structure of HMGCR was obtained from the Protein Data Bank (PDB ID: 1HMG). Before initiating docking simulations, water molecules were eliminated and polar hydrogen atoms were incorporated. Molecular docking was performed using AutoDock Vina. The binding site was defined as the catalytic active site of HMGCR, and a grid box was generated to cover the entire active site. The molecular configuration of Bru was obtained from PubChem. Subsequently, the HMGCR structure was converted into the PDBQT format, whereas Bru was prepared in the MOL2 format for docking simulations. A total of 100 docking simulations was executed in accordance with established protocols, and the simulation analysis was repeated three times to determine the binding affinity. The resultant data were visualised using PyMOL.

### 2.10 Surface plasmon resonance (SPR)

The SPR assay was conducted using a Biacore 1 K instrument, with HMGCR protein (50 μg/mL) immobilised on a CM5 sensor chip. Compound injections were performed at the indicated concentrations and flow rates. To obtain the final binding curves, solvent corrections were applied to the sensorgrams, as previously described (Guan et al., 2023).

### 2.11 Correlation of HMGCR expression with meningioma grade, recurrence, and cholesterol biosynthesis

The meningioma dataset mng_utoronto_2021 was acquired from cBioPortal (https://www.cbioportal.org/). The HMGCR mRNA levels were analysed in both high- and low-grade meningiomas using the ggplot2 package, with statistical significance determined through a two-tailed Student’s t-test. The association between HMGCR expression and tumour recurrence was evaluated using the Chi-square test.

### 2.12 Detection of cholesterol accumulation

Cells were treated with Bru for 48 h, with solvent-treated cells used as a vehicle control. After three washes with PBS, the cells were lysed, and the cholesterol levels were determined using a kit by measuring the absorbance at 570 nm.

The cells were fixed in 3% paraformaldehyde, washed three times with PBS, and treated with glycine to neutralise paraformaldehyde. Thereafter, the cells were stained with Filipin III and imaged using a fluorescence microscope to assess cholesterol accumulation.

### 2.13 Western blot analysis

Total protein was extracted using RIPA buffer from cultured cells treated with Bru at 0, 5, 10, and 20 nM. Equal amounts of proteins were electrophoresed on a sodium dodecyl sulphate-polyacrylamide gel and subsequently transferred onto a polyvinylidene fluoride membrane. After blocking, the membranes were incubated sequentially with primary antibodies and horseradish peroxidase (HRP)-conjugated secondary antibodies. The immunoblots were developed using an enhanced chemiluminescence reagent.

### 2.14 Transfection of small interfering RNAs (siRNAs)

IOMM-Lee cells (2 × 10^5^ cells per well) were seeded in 6-well plates, 24 h prior to transfection. In each well, 75 pmol of siHMGCR (HMGCR-si1: GGG​UAC​GUC​AGC​UUG​AAA​UTT; HMGCR-si2: GUC​CCA​GAC​AAU​UGU​UGU​ATT; HMGCR-si3: GCU​GGA​CGC​AAC​CUU​UAU​ATT; HMGCR-si4: GCA​GAU​UCU​AGC​CGU​UAG​UTT) was transfected into the cells using a 53.75 μL siRNA/siRNA-mate Plus complex. After transfection for 48 h, the cells were used for subsequent experiments.

### 2.15 Establishment of stable cell lines overexpressing HMGCR via lentiviral vector

The lentiviral construct designed for overexpressing HMGCR was synthesised by Nanjing Hengyi Biological Technology Co., Ltd (Nanjing, China). Lentiviral transfection was performed following the manufacturer’s protocols Positive cells were selected based on puromycin resistance, and the efficiency of transfection was assessed via Western blot analysis.

### 2.16 Animal and tumour xenograft model

All experimental procedures complied with the ethical guidelines of the Nanjing University of Chinese Medicine and received necessary authorisation (approval number: 202412A062). Eight-week-old BALB/c nude mice were employed to develop the tumor xenograft model and were housed in conditions free from specific pathogens. Cisplatin was selected as a positive control due to its established efficacy in preclinical models evaluating anti-cancer agents such as those used for meningioma (Dasari et al., 2014; Guo et al., 2020; Preusser et al., 2012). To establish the animal model, 5 × 10^6^ IOMM-Lee cells were mixed with Matrigel and injected subcutaneously into the right flank of BALB/c nude mice. All test agents were administered via intraperitoneal injection (i.p.) every other day. The mice were randomly allocated to six different groups (*n* = 6): control group; model group in which the mice received 0.1 mL normal saline for every 10 g of body weight daily; positive control group in which the mice were administered cisplatin (2 mg/kg, every other day), and three Bru-treated groups administered Bru (8, 4, and 2 mg/kg every other day), based on preliminary experiments and previous studies (Thompson et al., 2016; Yuan et al., 2025), with six mice per group. The mice were monitored for approximately 16 days, during which the body weight and tumour volume were recorded every 2 days. When the tumour diameter reached approximately 20 mm, the mice were euthanised, and tumour tissues were subsequently harvested, weighed, and photographed for further examination.

### 2.17 Haematoxylin and eosin (H&E) staining and immunohistochemistry (IHC)

The tumor samples were fixed in 4% paraformaldehyde, followed by a sequence of dehydration, xylene clearing, paraffin embedding, and sectioning processes. Paraffin sections were dewaxed sequentially and rinsed with tap water. Following staining, rinsing, differentiation, bluing, and running water rinsing, sections were dehydrated via gradient treatment, counterstained, then further dehydrated and cleared. Finally, sections were mounted for microscopic examination and image analysis.

For IHC, cryosections fixed with acetone were incubated with anti-Ki-67 (1:50, Abcam), HMGCR (1:1,000, Abmart), AKT (1:5,000, Proteintech), p-AKT (1:1,000, Abmart), PI3K p85 (1:5,000, Proteintech), or PI3K p85/p55 (1:5,000, Proteintech) antibody, diluted in DAKO diluent, for 60 min at room temperature. The sections were subsequently rinsed with PBS-Tween and then exposed to the relevant secondary antibody (anti-rabbit, Vector) for a duration of 30 min, and subsequently with avidin-biotin complex (ABC) for 30 min and AEC substrate for 3 min. The sections were counterstained with haematoxylin for 7 min. The presence of antibody-positive cells was subsequently evaluated via microscopic examination.

### 2.18 Statistical analysis

Data are expressed as mean ± standard deviation (mean ± SD) to reflect variability across three independent experimental replicates. Before applying parametric tests, we verified assumptions of normality and homogeneity of variance. Two-group comparisons utilized an unpaired two-tailed Student’s t-test, whereas one-way analysis of variance (ANOVA) was applied for datasets involving three or more groups. Statistical significance was defined as a *p*-value below 0.05. Furthermore, associations between evaluated variables were examined via the Spearman rank correlation test, a non-parametric approach suitable for data not following a normal distribution.

## 3 Results

### 3.1 Bru inhibits the proliferation, migration, and invasion but induces the apoptosis of meningioma cells

We first assessed the effect of Bru on the proliferation of IOMM-Lee and CH157-MN cells using the CCK-8 assay. Bru markedly suppressed the proliferation of both the cell lines in a concentration- and time-dependent manner. The IC_50_ of Bru for IOMM-Lee and CH157-MN was 22.84 and 30.19 nM at 24 h, 17.58 and 25.43 nM at 48 h, and 14.55 and 21.97 nM at 72 h, respectively (Figure 1B), highlighting its substantial potential for inhibiting meningioma development. These results were corroborated by those of colony formation assay, which showed that Bru significantly impeded the long-term growth of meningioma cells (Figure 1C). Additionally, EdU incorporation analysis showed a pronounced decrease in the proportion of EdU-positive meningioma cells following Bru treatment, indicative of its capability to inhibit DNA synthesis (Figure 1D).

**FIGURE 2:**
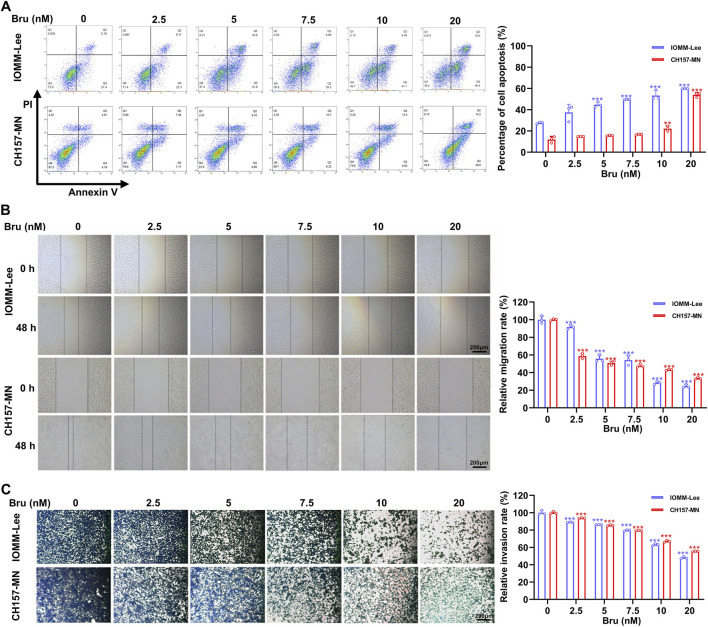
Brusatol (Bru) promoted apoptosis and inhibited the migration and invasion of meningioma cells **(A)** Bru promoted apoptosis of IOMM-Lee and CH157-MN cells, as evaluated using the Annexin V/propidium iodide staining assay **(B)** Bru inhibited the migration of IOMM-Lee and CH157-MN cells, as evaluated using the scratch assay **(C)** Bru inhibited invasion of IOMM-Lee and CH157-MN cells, as evaluated using the Transwell invasion assay. Data are presented as mean ± SD (*n* = 3); ^*^ *p* < 0.05, ^**^ *p* < 0.01, ^***^ *p* < 0.001 vs. the control group.

To further explore the pro-apoptotic effects of Bru in meningioma cells, the cells were treated with different concentrations of Bru. Flow cytometry analysis revealed an increase in apoptosis rates in Bru-treated meningioma cells, indicating effective triggering of apoptosis (Figure 2A).

As tumour recurrence is closely associated with the migration and invasion of tumour cells, we conducted scratch and Transwell assays to evaluate the effect of Bru on the migratory and invasive capabilities of meningioma cells. Across all tested concentrations, Bru significantly diminished the migration and invasion of IOMM-Lee and CH157-MN cells in a dose-dependent manner (Figures 2B,C). Collectively, these results indicated significant concentration-dependent anti-tumour effects of Bru on meningioma cells, characterised by pronounced inhibition of cell viability, proliferation, invasion,and migration, as well as a moderate pro-apoptotic effect.

### 3.2 Inhibitory effects of bru on meningioma cells are associated with cholesterol biosynthesis

**FIGURE 3:**
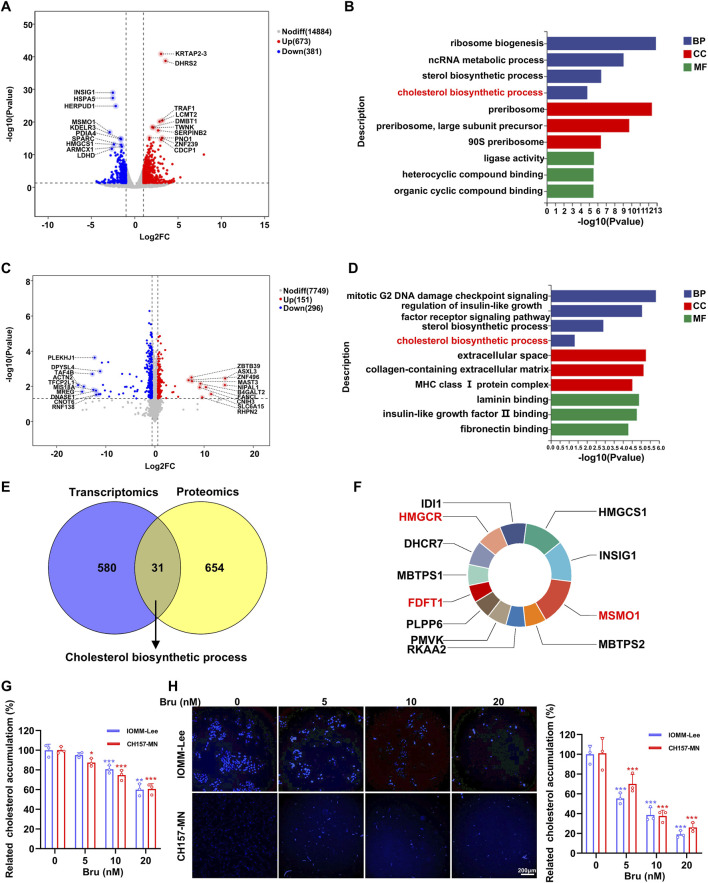
Transcriptomic and proteomic analyses of meningioma cells before and after Brusatol (Bru) treatment **(A)** Volcano plots displaying significantly different genes between the control and Bru group comparison in transcriptomic analysis **(B)** Gene ontology (GO) analysis of differentially expressed genes between control and Bru groups in transcriptomic analysis **(C)** Volcano plots showing differentially expressed proteins between control and Bru groups in proteomic analysis **(D)** GO analysis of differentially expressed proteins between control and Bru groups in proteomic analysis **(E)** Intersecting pathways with significant differences identified in both transcriptomic and proteomic analysis **(F)** Cholesterol biosynthesis genes showing significant differences are highlighted **(G)** Free cholesterol levels in Bru-treated IOMM-Lee and CH157-MN cells, quantified using a cholesterol assay kit **(H)** Free cholesterol levels in Bru-treated IOMM-Lee and CH157-MN cells, quantified using the Filipin III fluorescence staining method. Data are presented as mean ± SD (*n* = 3). ^*^ *p* < 0.05, ^**^ *p* < 0.01, ^***^ *p* < 0.001 vs. the control group.

To investigate the potential mechanisms about the inhibition of meningioma development by Bru, we performed transcriptomic and proteomic analyses on IOMM-Lee cells treated with 10 nmol/L Bru for 48 h. The transcriptomic analysis revealed a total of 1,054 differentially expressed genes (DEGs) in the Bru-treated vs. control group comparison, with 673 upregulated and 381 downregulated genes (Figure 3A). The GO enrichment analysis unveiled that Bru significantly influenced the genes primarily associated with biological processes (BP) based downregulated genes, including ribosome biogenesis, non-coding RNA metabolism, sterol biosynthetic pathway, and cholesterol biosynthesis (Figure 3B). The proteomic analysis identified 447 DEPs, which consisted of 151 upregulated and 296 downregulated proteins (Figure 3C). The GO analysis of the proteomic data revealed that the downregulated proteins were enriched in BP terms, including mitotic G2 DNA damage checkpoint pathway, regulation of insulin-like growth factor receptor signalling pathway, sterol biosynthetic processes, and cholesterol biosynthesis (Figure 3D).

Integrated multi-omics analysis entailed mapping transcriptomic and proteomic profiles to their shared gene identifiers. Only overlapping gene-protein pairs detected in both datasets were subjected to joint assessment. Expression consistency was defined as concordant downregulation at both the transcript and protein levels, and required a Pearson correlation coefficient greater than 0.6 for the corresponding fold-change values. Among the 31 GO terms, cholesterol biosynthesis process emerged as a significantly regulated by Bru (Figure 3E). 12 key DEPs were identified in cholesterol biosynthesis process, in which HMGCR, MSMO1, and FDFT1 were detected in both transcriptomic and proteomic datasets, whereas other 9 DEPs were exclusively identified in the transcriptomic data (Figure 3F). Therefore, HMGCR, MSMO1, and FDFT1 were considered to be key genes in cholesterol biosynthesis process regulated by Bru for further study.

To assess the effect on cholesterol biosynthesis, we evaluated cholesterol accumulation using a cholesterol assay kit and Filipin III fluorescence staining method, which revealed that Bru significantly inhibited cholesterol biosynthesis in both IOMM-Lee and CH157-MN cells in a concentration-dependent manner (Figures 3G,H). These findings indicate that the suppressed cholesterol biosynthesis may represent a fundamental mechanistic pathway through which Bru inhibits the biological behaviour of meningioma cells. This effect is possibly mediated through key genes, including HMGCR, MSMO1, and FDFT1.

### 3.3 HMGCR serves as a potential meningioma-specific marker that can be downregulated by bru

**FIGURE 4:**
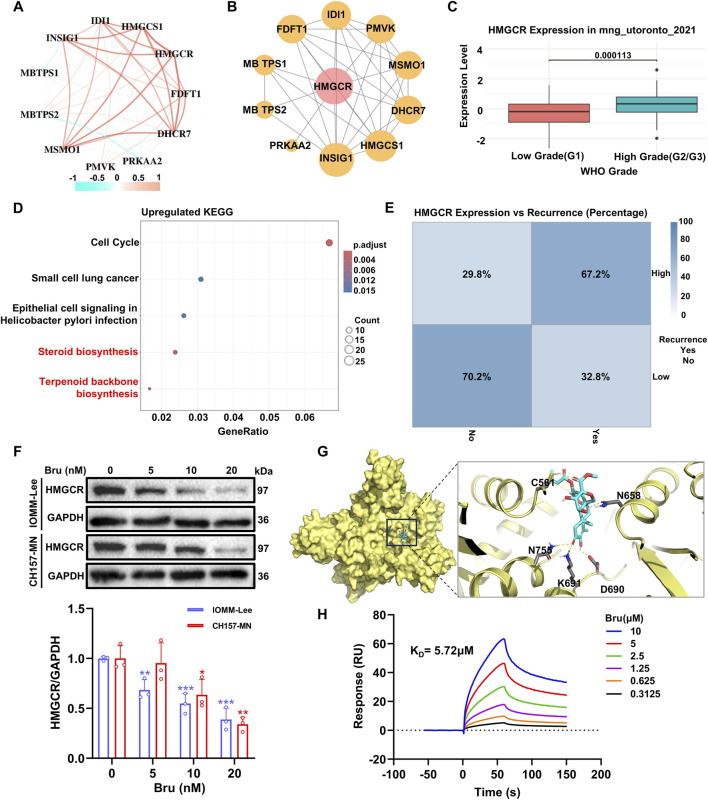
Integrated public database, Western blotting, molecular docking, and surface plasmon resonance analyses to dissect the key role of 3-hydroxy-3-methylglutaryl-CoA reductase (HMGCR) in the downregulation of the cholesterol biosynthesis pathway by Brusatol (Bru) **(A)** Correlation analysis of genes in the cholesterol biosynthesis pathway showed Pearson correlation coefficients with colour-coded intensities indicating positive and negative relationships **(B)** Protein interaction network among the cholesterol biosynthesis pathway genes illustrating their relationships **(C)** Comparison of HMGCR mRNA levels between high- and low-grade meningiomas in the mng_utoronto_2021 dataset **(D)** Kyoto encyclopedia of genes and genomes pathway analysis showing enrichment of upregulated genes in pathways associated with steroid biosynthesis **(E)** Correlation between HMGCR expression and meningioma recurrence **(F)** HMGCR protein levels in Bru-treated IOMM-Lee and CH157-MN cells, quantified and statistically analysed to evaluate the dose-dependent response **(G)** Molecular docking of Bru onto HMGCR **(H)** Surface plasmon resonance analysis for HMGCR and Bru. Data are presented as mean ± SD (*n* = 3). ^*^ *p* < 0.05, ^**^ *p* < 0.01, ^***^ *p* < 0.001 vs. the control group.

HMGCR is widely recognised as the principal rate-limiting enzyme involved in theintracellular synthesis of cholesterol. Unlike downstream enzymes MSMO1 and FDFT1, HMGCRs subject to multi-layered posttranslational regulation that controls the entire cholesterol synthesis. This central regulatory role makes it the most critical and informative target for our investigation (Schumacher & DeBose- Boyd, 2021). However, its involvement in the pathogenesis of meningioma has not yet been thoroughly investigated. To evaluate the potential use of HMGCR as a biomarker for meningioma, we conducted bioinformatics analyses employing public databases and experimental validation. As described in the previous section, a subsequent correlation analysis using 68 meningioma samples (GEO: GSE16581) revealed notable associations among MSMO1, FDFT1, and HMGCR in this biological context (Figure 4A). Protein–protein interaction analysis highlighted HMGCR as a pivotal gene in the cholesterol biosynthesis process (Figure 4B). To assess the clinical significance of HMGCR, we examined 121 meningioma cases from the cBioPortal (mng_utoronto_2021 dataset). HMGCR expression was significantly elevated in high-grade meningiomas compared with that in low-grade counterparts (Figure 4C). Furthermore, the KEGG pathway analysis of DEG categorised by HMGCR expression levels revealed substantial enrichment of cholesterol biosynthesis process consisting of steroid and terpenoid backbone biosynthesis pathway (Figure 4D). Moreover, the elevated expression of HMGCR correlated significantly with increased rates of tumour recurrence (Figure 4E).

Subsequently, we treated the cells with Bru and further assessed the HMGCR levels. A significant, dose-dependent reduction in the expression of HMGCR was observed for both the IOMM-Lee and CH157-MN cells treated with Bru (Figure 4F). Molecular docking analysis revealed that Bru interacted with HMGCR through multiple hydrogen bonds formed with the amino acid residues C561, N755, K691, D690, and N658 (Figure 4G). To further confirm the direct binding of Bru with HMGCR, we performed the SPR assay and evaluated the binding affinity. Bru was found to bind to HMGCR in a concentration-dependent manner, with a dissociation constant (*K*_D_) of 5.72 μM (Figure 4H). Collectively, these results suggest that HMGCR may serve as a tumour-specific marker for meningiomas and that Bru can downregulate its expression. This highlights the potential of targeting HMGCR with Bru in fighting against the progression of meningioma.

### 3.4 HMGCR promotes the progression of meningioma by enhancing cholesterol biosynthesis and PI3K-Akt pathway

**FIGURE 5:**
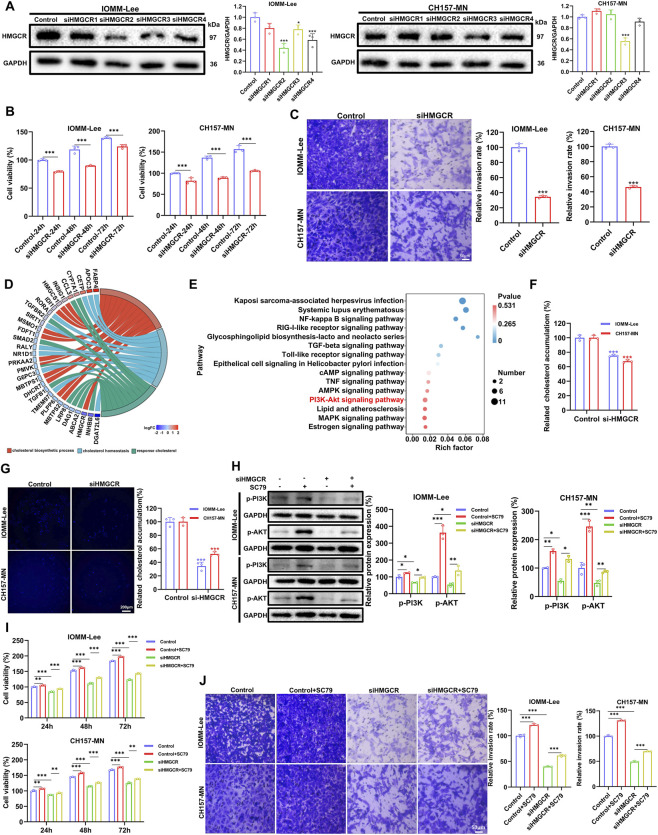
Function of 3-hydroxy-3-methylglutaryl-CoA reductase (HMGCR) and its relationship with the PI3K/AKT pathway in meningioma cells **(A)** HMGCR protein levels after knockdown in IOMM-Lee and CH157-MN cells assessed using Western blot **(B)** Viability of IOMM-Lee and CH157-MN cells after transfection with HMGCR siRNA or negative control siRNA, assessed using the cell counting kit-8 (CCK-8) assay at different time points **(C)** Transwell invasion assay of IOMM-Lee and CH157-MN cells after HMGCR knockdown **(D,E)** Gene ontology enrichment **(D)** and Kyoto encyclopedia of genes and genomes pathway **(E)** analyses illustrating gene enrichment terms associated with HMGCR knockdown in IOMM-Lee cells **(F)** Free cholesterol levels in HMGCR-knockdown cells compared with those in control cells, assessed using a cholesterol assay kit **(G)** Free cholesterol levels in HMGCR-knockdown cells compared with those in control cells, assessed using the Filipin III fluorescence staining method **(H)** Western blot analysis and corresponding statistical quantification of PI3K/AKT pathway-related proteins **(I)** Viability of HMGCR-knockdown IOMM-Lee and CH157-MN cells stimulated with SC79, assessed using the CCK-8 assay **(J)** Invasion of HMGCR-knockdown IOMM-Lee and CH157-MN cells stimulated with SC79, assessed using the Transwell assay. Data are presented as mean ± SD (*n* = 3). ^*^ *p* < 0.05, ^**^ *p* < 0.01, ^***^ *p* < 0.001.

To verify our bioinformatic prediction regarding the role of HMGCR in meningioma, we investigated the effects of HMGCR knockdown on cholesterol levels and tumourigenic properties of meningioma cells. In particular, we evaluated cell growth and invasion using the CCK-8 and Transwell invasion assays. Western blot analysis confirmed the effective knockdown of HMGCR with siRNA, showing a significant decrease in the protein levels in the IOMM-Lee and CH157-MN cells when compared to that in cells transfected with the empty vector (EV) control (Figure 5A). Further analyses indicated that HMGCR knockdown substantially inhibited the growth and invasion of meningioma cells (Figures 5B,C).

To gain deeper insights into the downstream mechanisms mediating the pro-tumourigenic property of HMGCR, we performed transcriptome sequencing following siRNA-mediated HMGCR knockdown. Subsequent KEGG enrichment analyses revealed strong association of HMGCR expression with two distinct pathways—the cholesterol biosynthesis and PI3K-Akt pathways (Figures 5D,E). We further measured cholesterol levels as well as conducted rescue experiments targeting PI3K-AKT to validate the findings of the omics analyses. As a result, after HMGCR knockdown, cholesterol levels of cells was substantially inhibited (Figures 5F,G). HMGCR knockdown inhibited the phosphorylation of both AKT and PI3K, and these effects were reversed upon treatment with the Akt activator SC79 (Figure 5H). Notably, SC79 treatment significantly alleviated the suppressed growth and invasion of meningioma cells mediated through HMGCR siRNA (Figures 5I,J).

Together with the experimental findings described in the previous section, these results establish HMGCR as a key regulator of meningioma progression, which is essential for enhancing the growth and invasion of tumour cells. As a key mediator of cholesterol biosynthesis in meningioma cells, HMGCR drives malignant phenotypes by activating the cholesterol biosynthesis and PI3K-Akt signalling pathway, thereby facilitating tumour growth and invasion.

### 3.5 Bru represses the progression of meningioma via restricting the cholesterol biosynthesis and inhibiting the PI3K-Akt pathway

Based on preliminary mechanistic studies, we surmised that Bru could inhibit the progression of meningioma by down-regulating the expression of HMGCR, which might in turn decrease the cholesterol level and suppress the activation of the PI3K/AKT signalling pathway. To validate this hypothesis, we performed a comprehensive pharmacodynamic analysis.

**FIGURE 6:**
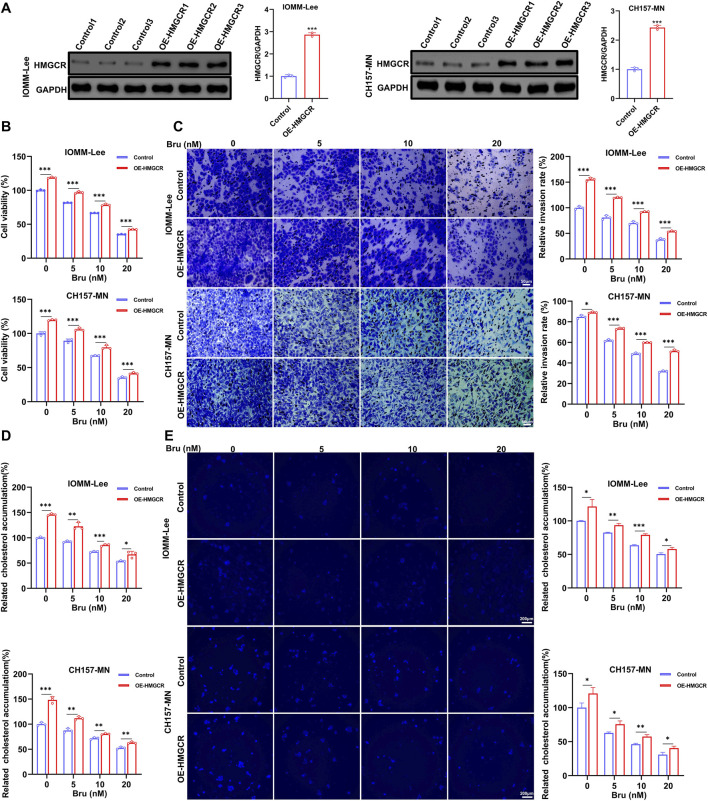
3-Hydroxy-3-methylglutaryl-CoA reductase (HMGCR) overexpression reverses the anticancer effects of Brusatol (Bru) **(A)** Western blots showing successful generation of HMGCR-overexpressing cell lines **(B)** Viability of IOMM-Lee and CH157-MN cells transfected with HMGCR overexpression or empty vector (EV) after 48 h of Bru treatment, assessed using the cell counting kit-8 assay **(C)** Effect of Bru on the invasion of IOMM-Lee and CH157-MN cells transfected with EV or overexpressing HMGCR, assessed using the Transwell assay **(D)** Free cholesterol levels in IOMM-Lee and CH157-MN cells transfected with EV or overexpressing HMGCR, quantified using a cholesterol assay kit **(E)** Free cholesterol levels in IOMM-Lee and CH157-MN cells after Bru treatment in control and HMGCR-overexpressing groups, quantified using the Filipin III fluorescence staining method. Data are presented as mean ± SD (*n* = 3). ^*^ *p* < 0.05, ^**^ *p* < 0.01, ^***^ *p* < 0.001, vs. the control group.

To substantiate the hypothesis that Bru alleviates the progression of meningioma via the modulation of HMGCR, we developed stable 1OMM-Lee and CH157-MN cell models of HMGCR overexpression. Overexpression of HMGCR in the cells was confirmed via Western blot analysis, with significantly increased HMGCR protein levels observed compared with those in the EV control group (Figure 6A). Notably, the inhibitory effects of Bru on cellular growth, invasion, and cholesterol biosynthesis were partially reversed by HMGCR overexpression. These results support the contention that HMGCR plays a crucial role in mediating the anti-meningioma efficacy of Bru (Figures 6B–E).

**FIGURE 7:**
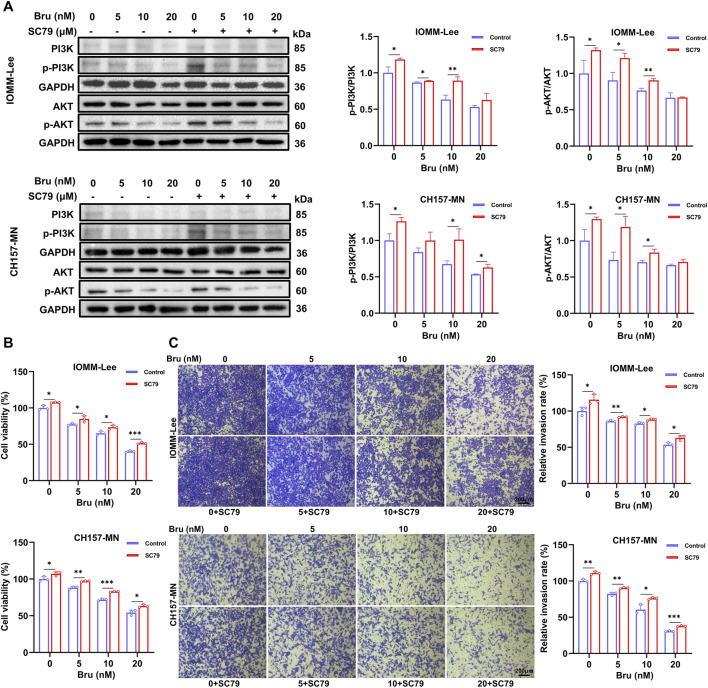
Effects of Brusatol (Bru) concentration on PI3K/AKT protein expression, cell viability, and invasion in SC79-pretreated IOMM-Lee and CH157-MN cells subjected to rescue experiments **(A)** Expression levels and quantitative analysis of PI3K/AKT proteins in IOMM-Lee and CH157-MN cells **(B)** Viability of Bru-treated IOMM-Lee and CH157-MN cells, assessed using the cell counting kit-8 assay **(C)** Invasion of IOMM-Lee and CH157-MN cells treated with different concentrations of Bru, assessed using the Transwell invasion assay. Data are presented as mean ± SD (*n* = 3). Statistical significance is indicated as ^*^ *p* < 0.05, ^**^ *p* < 0.01, ^***^ *p* < 0.001, vs. the control group.

Further mechanistic investigations were carried out to verified whether the inhibitory effect of Bru on the phosphorylation levels of PI3K/AKT closely mirrored the effects observed for siRNA-mediated HMGCR knockdown, as described in “Section 3.5”. Rescue experiments were performed using the AKT activator SC79 in the IOMM-Lee and CH157-MN cells. Bru significantly inhibited the phosphorylation levels of PI3K and AKT in a dose-dependent manner, which could be reversed by SC79 (Figure 7A). Notably, SC79 also mitigated Bru-induced inhibition of the growth and invasion of meningioma cells (Figures 7B,C). Collectively, these findings indicate that Bru inhibits the progression of meningioma via down-regulating HMGCR, leading to decreased cholesterol biosynthesis and suppressed PI3K/AKT signalling cascade.

### 3.6 Bru suppresses the progression of meningioma *in vivo*

**FIGURE 8:**
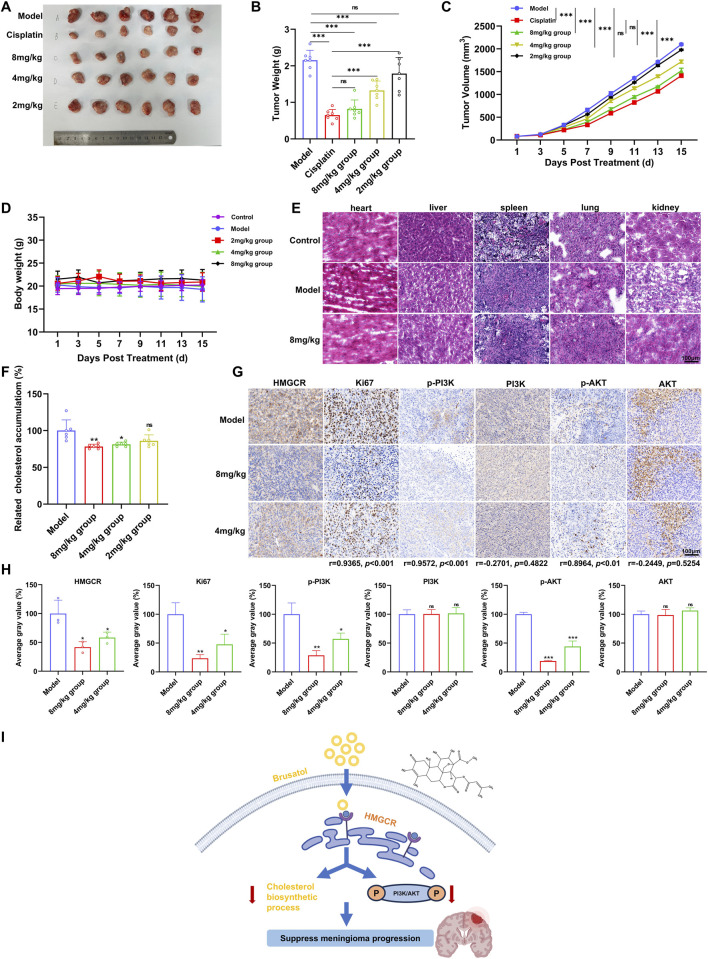
Anti-tumour effects of Brusatol (Bru) in BALB/c nude mice model of meningioma and the underlying molecular mechanism **(A)** Representative images of subcutaneous meningioma tissues from nude mice treated with 2, 4, or 8 mg/kg of Bru, cisplatin, or normal saline (*n* = 6) **(B)** Tumour weights measured at the time of sample collection **(C)** Tumour growth curves for mice over 15 days **(D)** Body weight of mice was monitored every 2 days throughout the study **(E)** Histopathological examination of haematoxylin and eosin-stained heart, liver, spleen, lung, and kidney sections **(F)** Bru reduced the free cholesterol content in tumour tissues **(G)** Immunohistochemical analysis of the expression of proliferation markers HMGCR, Ki-67, p-PI3K, PI3K, p-AKT and AKT in tumour tissues from model mice and mice treated with 4 or 8 mg/kg of Bru **(H)** Statistical analysis of immunohistochemistry results **(I)** Schematic illustration of the cancer-inhibiting mechanisms of Bru in meningioma cells. Data are presented as mean ± SD (*n* = 3). ^*^ *p* < 0.05, ^**^ *p* < 0.01, ^***^ *p* < 0.001. Ns indicates no statistically significant difference.

To further substantiate the anti-tumour efficacy of Bru and to elucidate its underlying mechanisms *in vivo*, a meningioma xenograft model was established on BALB/c nude mice. Bru treatment led to a dose-dependent inhibition of tumour growth when compared to that in the model group treatment (Figure 8A). In particular, 4 and 8 mg/kg/day of Bru resulted in the reduction of tumour weight by 38% and 62%, respectively, together with a dramatic decrease in tumour volume (*p* < 0.001 vs. the model group) (Figures 8B,C). Both Brusatol (8 mg/kg) and cisplatin significantly reduced tumour weight and volume, with no statistically significant differences observed between the two groups at endpoint (*p* > 0.05). More specifically, the reductions in tumor weight and volume of Bru group at the doses of 8 mg/kg/day exhibited no significant difference when compared to those of cisplatin group, indicating that the anti-meningioma effect of Bru can reach the level of positive drugs (Figures 8A,B). Monitoring of body weight across different treatment groups revealed that Bru had no significant toxicity or adverse effects at the administered doses during the study period (*p* > 0.05) (Figure 8D). This finding was further substantiated by histopathological assessment of vital organs, including the liver, kidney, heart, lung, and spleen (Figure 8E). However, the relatively short duration of the animal experiment limits conclusions regarding long-term safety. Moreover, cholesterol levels in the Bru-treated mice exhibited considerable reduction in a dose-dependent manner (Figure 8F). These findings collectively indicate that Bru effectively retards tumour progression without exhibiting significant toxicity at the administrated doses ranging from 2 to 8 mg/kg/day.

IHC analysis revealed that Bru downregulated the expression of HMGCR and inhibited the PI3K/AKT signalling pathway. Remarkably, the protein levels of HMGCR, p-AKT, and p-PI3K were markedly reduced, whereas the total AKT and total PI3K levels remained unaffected following Bru treatment (Figures 8G,H). Besides, Ki-67 immunohistochemical staining of model group revealed a high proliferative index of 30%–35%, consistent with the malignant characteristics of high-grade meningioma. Notably, Furthermore, a positive correlation between HMGCR expression and proliferation of meningioma cells was evidenced by Ki-67 IHC staining of meningioma tissue samples. The Pearson correlation coefficient was calculated and the *r* value was determined to be 0.9365 (*p* < 0.001). With increasing Bru concentration, a corresponding decrease in the expression of HMGCR was noted, which was associated with reduced levels of p-PI3K and p-AKT. Pearson correlation analysis further showed a positive correlation between HMGCR and pivotal proteins involved in the PI3K/AKT signalling pathway. The correlation coefficient *r* was 0.9572 for p-PI3K (*p* < 0.001) and 0.8964 for p-AKT (*p* < 0.01).

In summary, all the results of the whole experiment indicate that Bru inhibits tumour progression by targeting HMGCR to restrict the cholesterol biosynthesis and modulate the PI3K/AKT signalling pathway (Figure 8I).

## 4 Discussion

In the present study, we found that Bru significantly restricts cholesterol biosynthesis through downregulating the expression level of HMGCR. The decreased expression of HMGCR subsequently interfered with the activation of the PI3K/AKT signaling pathway, leading to the inhibition on proliferation of meningioma cells. This study represented the first attempt to elucidate the mechanisms underlying Bru limited meningioma progression by influencing cholesterol metabolism and the PI3K/AKT signaling pathway. The results provided novel insights into promising therapeutic strategies for meningioma.

Additionally, our *in vitro* data revealed that Bru effectively suppressed the cellular proliferation, triggered apoptosis, and reduced the invasion and migration of meningioma cells, highlighting its potential as a treatment to combat the metastasis of meningioma. The dose-dependent inhibitory effects of Bru on the malignant characteristics of meningioma cells align with previous research findings pertaining to various other cancers, such as non-small cell lung carcinoma, hepatocellular carcinoma, and colorectal carcinoma. (Cai et al., 2019; Xi et al., 2024). Notably, Bru was significantly effective against meningioma cells, with IC_50_ values ranging from 14.55 to 30.19 nM, underscoring its robust therapeutic potential. Furthermore, the *in vivo* experiments showed substantial dose-dependent tumour suppression (38%–62% reduction) at the administered doses (four to eight mg/kg/day) without observable toxicity. Most of importance, the reductions in tumour weight and volume of Bru’s group at the administered doses of 8 mg/kg/day exhibited no significant difference when compared to those of cisplatin group, indicating a favourable efficacy and safety profile of Bru. These results were similar to the effects observed in other tumors. (Huang et al., 2023). Collectively, these findings implied the clinical potential of Bru for meningioma treatment.

The comprehensive transcriptomic and proteomic analyses suggested that Bru might downregulate cancer-related pathways, particularly those involved in cholesterol biosynthesis and the enzyme including HMGCR, MSMO1 and FDFT1, which have been reported to play pivotal roles in tumour progression via influencing cellular survival, proliferation, motility, and metabolic reprogramming (Xiao et al., 2023). These suggestions were partly validated by molecular docking, SPR analysis, and assessment of protein and cholesterol levels. While most previous studies focused on the Wnt/β-catenin and Hedgehog signaling pathways, cholesterol biosynthesis and HMGCR have frequently been overlooked in the context of meningioma (Okano et al., 2022; Suppiah et al., 2019). Since HMGCR is a pivotal rate-limiting enzyme in cholesterol synthesis process, which can catalyze the conversion of HMG-CoA to mevalonate (Xue et al., 2020), further bioinformatics analyses from GEO: GSE16581 and cBioPortal (mng_utoronto_2021 dataset) revealed that there were significant correlation and interactions between the expression of HMGCR and *MSMO1* or FDFT1. HMGCR was significantly elevated in high-grade meningiomas. HMGCR was enriched in cholesterol biosynthesis pathways, and its expression was correlated with higher tumor recurrence rates. Previous studies have established a strong correlation between elevated HMGCR expression and poor prognosis across multiple cancer types, including breast and colorectal cancer (Bengtsson et al., 2014; Borgquist et al., 2008). Aberrant overexpression of HMGCR disrupts cholesterol homeostasis and impairs membrane fluidity, metabolic stability, and signal transduction, subsequently accelerate tumour cell growth and invasiveness across different types of cancers (Clendening et al., 2010; Mullen et al., 2016). All the bioinformatic results were further validated using experiments, wherein HMGCR knockdown led to significant decrease in the growth and migration of tumour cells, as well as inhibition of cholesterol biosynthesis. Notably, the inhibitory effects of Bru on cellular growth, invasion, and cholesterol biosynthesis were partially reversed by HMGCR overexpression. The results also verified in a nude mice model of meningioma. All these results support the hypothesis that HMGCR plays a crucial role in Bru’s anti-meningioma efficacy as well as in the pathogenesis of meningioma.

The PI3K/Akt signaling pathway, which is involved in various types of cancers, exerts considerable effects on the tumour microenvironment, particularly on angiogenesis and recruitment of inflammatory mediators (He et al., 2021). PI3K/Akt signalling pathway is commonly dysregulated in high-grade meningiomas, contributing to tumorigenesis through enhanced cell proliferation and survival. This aberrant activation promotes meningioma progression and is linked to aggressive tumor behavior. Targeting this pathway may offer therapeutic approaches for meningioma treatment. (Li et al., 2023; Dunn et al., 2019). In present study, the downstream mechanisms mediating the pro-tumorigenic effects of HMGCR, using transcriptome sequencing following HMGCR knockdown revealed strong association of HMGCR expression with two distinct pathways—the cholesterol biosynthesis and PI3K-Akt pathways. Specifically, HMGCR knockdown inhibited the phosphorylation of Akt and PI3K, which could be reversed by SC79, as demonstrated by assays examining cellular growth and invasion. Consistent findings were also observed in the Bru -treated cells and partly verified by the *in vivo* experiment. Previous studies have indicated a potential impact of the biosynthesis of cholesterol or HMGCR expression in the PI3K/Akt signaling pathway. Beyond cholesterol production, HMGCR is also responsible for the synthesis of isoprenoids, including farnesyl pyrophosphate and geranylgeranyl pyrophosphate. These isoprenoids are important for the prenylation of various G-proteins and their subunits, such as members of the Ras and Rho super-families, which are crucial for the transduction of intracellular signalling and exhibit a direct correlation with the PI3K/Akt signalling pathway (Demierre et al., 2005). Notably, repression of HMGCR inhibits the PI3K/Akt signalling pathway. For instance, Wu et al. demonstrated that cholesterol activated the PI3K/Akt signalling pathway, which in turn facilitated the progression of colorectal cancer (Wu et al., 2022). In addition, Iizuka-Ohashi et al. reported that inhibition of the MVA pathway by HMGCR inhibitors mitigated the resistance against MEK inhibitor-induced apoptosis by restricting the activation of the Akt signalling pathway in cancer cells (Iizuka-Ohashi et al., 2018). Therefore, HMGCR may represent a promising therapeutic target for meningiomas due to its impact on cholesterol biosynthesis and the PI3K/Akt pathway.

Recent studies have shown that Bru possesses prominent anti-tumor activity against various types of tumors, including neuroendocrine tumors, gliomas, hematologic malignancies, and nasopharyngeal carcinoma, through regulating the pathways including epithelial-mesenchymal transition, Nrf2, and PI3K/Akt/mTOR signaling pathways. Mechanism of action of Bru in different tumors may vary depending on the specific type of cancer. Meanwhile, there is currently a lack of research exploring the mechanism of action of Bru in meningioma. Our study provides novel insights into the anticancer effects of Bru on meningioma, as it appears to targeting HMGCR to regulate cholesterol synthesis and the PI3K/AKT axis, as validated by both *in vivo* and *in vitro* experiments. The anti-meningioma activity of Bru and its mechanisms of action involving HMGCR and cholesterol biosynthesis pathway had not been previously documented (He et al., 2023), which may offer a new avenue for exploring its underlying mechanisms of action. Importantly, our findings do not suggest that Brusatol should simply be regarded as another HMGCR inhibitor. Rather, this study identifies HMGCR as a previously unrecognized molecular target that mediates the antitumor activity of Brusatol in meningioma. Given the reported pleiotropic biological effects of Brusatol, additional mechanisms may act synergistically with HMGCR inhibition and warrant further investigation. Future studies comparing Brusatol with clinically approved statins and evaluating potential combination strategies will elucidate its therapeutic value more comprehensively.

This study has several limitations. First, while the IOMM-Lee xenograft model is widely used for high-grade meningioma research, it does not fully capture the biological heterogeneity of benign meningiomas; thus, future work should use patient-derived organoids to further validate the translational relevance of our findings. Second, cancer progression involves complex biological processes, and although Brusatol (Bru) markedly inhibits tumor growth and invasion, its underlying mechanisms warrant deeper investigation. Looking ahead, future research should assess the safety and efficacy of Bru across different meningioma subtypes, explore mechanisms of resistance, and develop combination strategies with conventional therapies. Additionally, systematic comparisons between Bru and statins, HMGCR inhibitors—focusing on antitumor efficacy, multi-target synergy, and toxicology—are needed to evaluate the potential of combination therapy.

## 5 Conclusion

This study demonstrates the potent anti-meningioma effects of Bru via selective targeting of cholesterol metabolism, revealing that this potential mechanism is distinct from its action on other malignancies. We found that Bru suppresses the progression of meningioma by targeting HMGCR to downregulate cholesterol biosynthesis and inhibit the PI3K/AKT signalling pathway—a novel mechanism that provides avenues for novel therapeutic strategies for targeted metabolic intervention in meningioma. Bru shows excellent efficacy and a favourable safety profile, supporting its further development as a novel therapeutic strategy for patients with meningioma.

## Data Availability

The data presented in the study are openly available in the Zenodo repository under the accession number 20748177, https://doi.org/10.5281/zenodo.20748177; accession number 20754662, https://doi.org/10.5281/zenodo.20754662.
