# Supplementary material for: Brusatol suppresses meningioma progression via targeting HMGCR to restrict the cholesterol biosynthesis and inhibit PI3K/AKT signaling pathway
Source: Front Pharmacol. 2026 Jun 30;17:1864031. doi: 10.3389/fphar.2026.1864031 (PMC13364563; doi:10.3389/fphar.2026.1864031)
Supplement: Supplementary file 1 [file DataSheet1.pdf]

Effect of Brusatol on Cell Cycle Distribution in IOMM-Lee and CH157-MN Cells: Analysis by Flow Cytometry

Methods

IOMM-Lee and CH157-MN cells were treated with Brusatol (0, 10, and 20 nM) for 48 h. Cells were harvested using 0.25% trypsin and centrifuged at 3000 × g for 2 min. After being washed twice with PBS, cells were fixed overnight at 4 °C in ice-cold 100% ethanol. Cells were subsequently incubated with propidium iodide (PI) staining solution for 30 min at 37°C in the dark using the Cell Cycle and Apoptosis Analysis Kit (Beyotime Biotechnology, Shanghai, China). Cell cycle distribution was then assessed using a Gallios flow cytometer, and the resulting data were analyzed using Kaluza software.

Results

Flow cytometric analysis showed that treatment with Brusatol at concentrations of 10 and 20 nM for 48 h did not significantly affect cell cycle distribution in either IOMM-Lee or CH157-MN cells **(Figure S1)**. The proportions of cells in the G1, S, and G2/M phases were comparable to those in the control group.

Conclusion

These findings indicate that Brusatol does not induce detectable cell cycle arrest in IOMM-Lee or CH157-MN cells under the experimental conditions tested. Therefore, the growth-inhibitory effect of Brusatol is likely attributable to apoptosis induction rather than alterations in cell cycle progression.

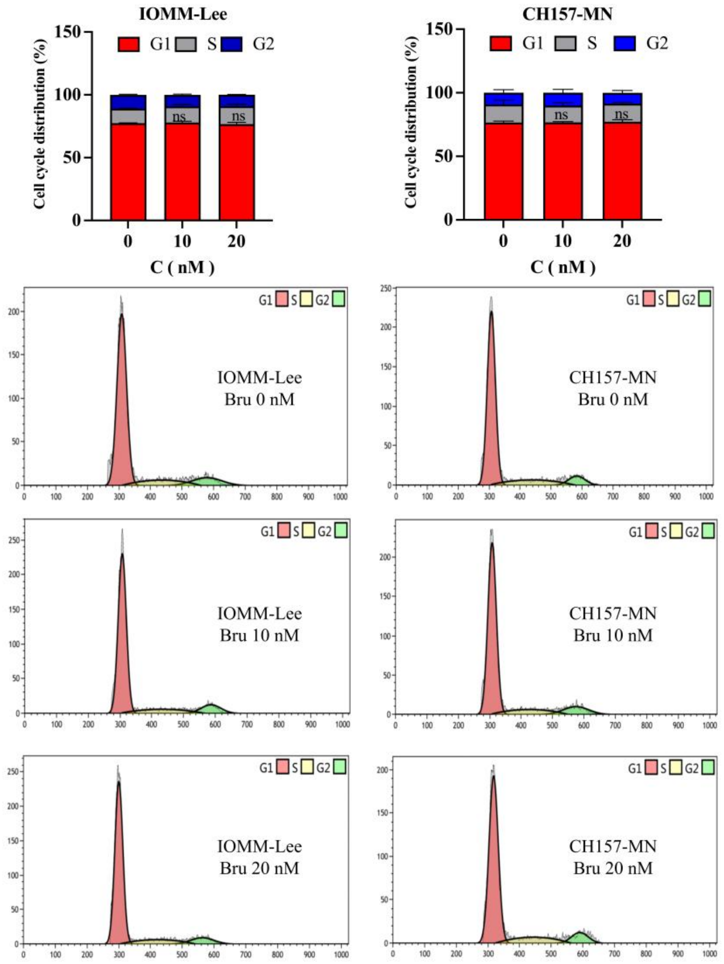

Figure S1. Cell cycle distribution of IOMM-Lee and CH157-MN cells after treatment with 0, 10, and 20 nM Brusatol for 48 h, determined by flow cytometry. Data are presented as mean ± SD (*n* = 3). $^{*}p < 0.05$; $^{**}p < 0.01$; $^{***}p < 0.001$ *vs*. the control group.
